# Supplementary material for: MetaRibo-Seq measures translation in microbiomes
Source: Nat Commun. 2020 Jun 29;11:3268. doi: 10.1038/s41467-020-17081-z (PMC7324362; doi:10.1038/s41467-020-17081-z)
Supplement: Supplementary file 10 — Supplementary Data 7 [file 41467_2020_17081_MOESM10_ESM.zip › File2/Confidence_High_Taxonomy/199686_out.krona.html]

Javascript must be enabled to view this page.

members
magnitude
magnitudeUnassigned
count
unassigned
taxon
rank

199686\_out

235

235
2
superkingdom

235
976
phylum

235
200643
class

235
171549

SRS013158\_contig\_number\_166SRS013800\_contig\_number\_18945SRS016541\_contig\_number\_14053SRS017375\_contig\_number\_485SRS017821\_contig\_number\_30246SRS019068\_contig\_number\_65931SRS023971\_contig\_number\_325SRS045004\_contig\_number\_6626SRS047433\_contig\_number\_contig-100\_900.128182SRS054352\_contig\_number\_13638SRS057478\_contig\_number\_5759SRS062427\_contig\_number\_10380SRS065397\_contig\_number\_contig-100\_888.82615SRS077024\_contig\_number\_10300SRS077194\_contig\_number\_113SRS077231\_contig\_number\_23506SRS078257\_contig\_number\_contig-100\_7141.7141SRS1041136\_contig\_number\_20767SRS148874\_contig\_number\_contig-100\_1067.98438SRS893383\_contig\_number\_26580
order
20


SRS893380\_contig\_number\_1060
1
species
1950538

family
815
156

49
genus

SRS011084\_contig\_number\_17378SRS012902\_contig\_number\_1371SRS012969\_contig\_number\_29654SRS013687\_contig\_number\_32842SRS014855\_contig\_number\_11379SRS015431\_contig\_number\_83098SRS015782\_contig\_number\_contig-100\_768.250409SRS017103\_contig\_number\_36960SRS017307\_contig\_number\_25122SRS018351\_contig\_number\_14353SRS018623\_contig\_number\_19527SRS019968\_contig\_number\_14785SRS020508\_contig\_number\_contig-100\_181.21170SRS023526\_contig\_number\_contig-100\_710.230363SRS023829\_contig\_number\_1025SRS024331\_contig\_number\_32414SRS024435\_contig\_number\_12760SRS045195\_contig\_number\_8230SRS046068\_contig\_number\_contig-100\_561.42243SRS046369\_contig\_number\_14258SRS049446\_contig\_number\_410SRS050752\_contig\_number\_contig-100\_811.169681SRS053335\_contig\_number\_377SRS053398\_contig\_number\_contig-100\_658.163792SRS053649\_contig\_number\_1369SRS056259\_contig\_number\_27264SRS057480\_contig\_number\_contig-100\_218.80878SRS063190\_contig\_number\_11892SRS063813\_contig\_number\_contig-100\_413.57644SRS075773\_contig\_number\_32434SRS077127\_contig\_number\_15506SRS077641\_contig\_number\_contig-100\_577.130653SRS077849\_contig\_number\_25661SRS097920\_contig\_number\_26700SRS098061\_contig\_number\_135SRS098620\_contig\_number\_contig-100\_1270.72778SRS098644\_contig\_number\_24315SRS098655\_contig\_number\_9127SRS1041031\_contig\_number\_contig-100\_501.155878SRS144506\_contig\_number\_contig-100\_136.321160SRS144537\_contig\_number\_10874SRS144603\_contig\_number\_13021SRS145308\_contig\_number\_4291SRS149075\_contig\_number\_4274SRS149325\_contig\_number\_14969SRS149784\_contig\_number\_contig-100\_706.167308SRS893259\_contig\_number\_6129SRS893279\_contig\_number\_12765SRS893288\_contig\_number\_2047
156
816


SRS013965\_contig\_number\_36037SRS016753\_contig\_number\_4695SRS017745\_contig\_number\_contig-100\_186.39697SRS020328\_contig\_number\_35489SRS021484\_contig\_number\_25056SRS042966\_contig\_number\_13866SRS052576\_contig\_number\_234SRS1041039\_contig\_number\_contig-100\_712.151789SRS1041116\_contig\_number\_9331SRS1041133\_contig\_number\_27320SRS1041137\_contig\_number\_1822SRS1055056\_contig\_number\_contig-100\_179.83019SRS893295\_contig\_number\_13141
species
47678
13


SRS011302\_contig\_number\_11396
1638779
species
1


SRS098073\_contig\_number\_17491
1
species
371601


SRS893366\_contig\_number\_17701
329854
species
1


SRS047044\_contig\_number\_376
1
species
291644


SRS014235\_contig\_number\_12470SRS015663\_contig\_number\_33589SRS016954\_contig\_number\_22537SRS020394\_contig\_number\_12406SRS024132\_contig\_number\_8915SRS024625\_contig\_number\_contig-100\_690.110376SRS048060\_contig\_number\_13592SRS064757\_contig\_number\_20032SRS065504\_contig\_number\_38574SRS075963\_contig\_number\_contig-100\_96.22806SRS1041118\_contig\_number\_3872SRS1041132\_contig\_number\_1119SRS1054691\_contig\_number\_20281SRS1055069\_contig\_number\_7540SRS147557\_contig\_number\_31333SRS148721\_contig\_number\_18607
16
1896974
species

species
1739517
1

SRS105153\_contig\_number\_7632


SRS014534\_contig\_number\_2508SRS016381\_contig\_number\_contig-100\_742.30347SRS043768\_contig\_number\_32015SRS045645\_contig\_number\_13422SRS048164\_contig\_number\_1424SRS063489\_contig\_number\_contig-100\_390.59484SRS064232\_contig\_number\_3439SRS1041091\_contig\_number\_contig-100\_912.120729SRS1041138\_contig\_number\_contig-100\_1327.72622SRS1041157\_contig\_number\_contig-100\_2272.48277SRS148091\_contig\_number\_10978
820
species
11


SRS056273\_contig\_number\_11102
1
1262739
species

species
46506
1

SRS016203\_contig\_number\_11843


SRS1041134\_contig\_number\_109
1
species
1796613

species
457387
22

SRS012273\_contig\_number\_contig-100\_986.266191SRS012849\_contig\_number\_13445SRS014923\_contig\_number\_24558SRS017191\_contig\_number\_18172SRS017622\_contig\_number\_583SRS019445\_contig\_number\_31422SRS019496\_contig\_number\_17161SRS020271\_contig\_number\_3926SRS022071\_contig\_number\_32732SRS022609\_contig\_number\_44038SRS043667\_contig\_number\_1780SRS043701\_contig\_number\_1418SRS049896\_contig\_number\_31914SRS053087\_contig\_number\_751SRS053356\_contig\_number\_44280SRS063370\_contig\_number\_360SRS078419\_contig\_number\_31359SRS098827\_contig\_number\_300SRS104975\_contig\_number\_17365SRS893172\_contig\_number\_contig-100\_1129.80732SRS893187\_contig\_number\_contig-100\_981.104756SRS893342\_contig\_number\_contig-100\_939.67526

species
821
4

SRS018836\_contig\_number\_666SRS044535\_contig\_number\_1447SRS075984\_contig\_number\_contig-100\_642.162238SRS148817\_contig\_number\_14086


SRS893341\_contig\_number\_6105
1
species
469590

1841855
species
2

SRS893292\_contig\_number\_contig-100\_270.66031SRS893300\_contig\_number\_333


SRS014287\_contig\_number\_19223SRS016989\_contig\_number\_1069SRS043841\_contig\_number\_contig-100\_872.76903SRS045244\_contig\_number\_7120SRS058268\_contig\_number\_244SRS062654\_contig\_number\_17921SRS075078\_contig\_number\_9590SRS1041140\_contig\_number\_17389
species
817
8


SRS024075\_contig\_number\_9570
species
2292943
1


SRS049959\_contig\_number\_13247SRS057717\_contig\_number\_1222SRS077730\_contig\_number\_1011SRS142980\_contig\_number\_272SRS143372\_contig\_number\_3101SRS147614\_contig\_number\_2824SRS148511\_contig\_number\_680
7
species
28116


SRS016132\_contig\_number\_80SRS021219\_contig\_number\_7909SRS1041038\_contig\_number\_contig-100\_52.106045SRS1055095\_contig\_number\_1334
4
species
2302926


SRS019285\_contig\_number\_contig-100\_1.21891
1
291645
species

9
species
246787

SRS045739\_contig\_number\_181SRS074670\_contig\_number\_31SRS076756\_contig\_number\_16605SRS1041036\_contig\_number\_428SRS1041129\_contig\_number\_2528SRS1054928\_contig\_number\_4005SRS144753\_contig\_number\_12279SRS893358\_contig\_number\_5156SRS893378\_contig\_number\_contig-100\_227.143245

2005473
family
10

1807756
species
10

SRS015590\_contig\_number\_15122SRS016335\_contig\_number\_10617SRS016495\_contig\_number\_15689SRS018817\_contig\_number\_24574SRS047014\_contig\_number\_21277SRS097889\_contig\_number\_27361SRS098881\_contig\_number\_17990SRS101376\_contig\_number\_20442SRS142890\_contig\_number\_34006SRS143417\_contig\_number\_24462

7
family
171550

7
genus
239759


SRS018936\_contig\_number\_11224SRS050026\_contig\_number\_8197
species
328814
2

2
species
2292190

SRS024549\_contig\_number\_96SRS893382\_contig\_number\_431


SRS050998\_contig\_number\_17370
1
28117
species


SRS053214\_contig\_number\_168
1
species
1872444


SRS074586\_contig\_number\_5645
species
2292910
1

2005525
family
35

genus
5
375288
35

SRS013521\_contig\_number\_14239SRS017701\_contig\_number\_contig-100\_1407.119920SRS078242\_contig\_number\_25105SRS078260\_contig\_number\_contig-100\_1090.26208SRS145497\_contig\_number\_19634


SRS014613\_contig\_number\_17276SRS015794\_contig\_number\_11666SRS017433\_contig\_number\_15960SRS019381\_contig\_number\_17218SRS023346\_contig\_number\_1527SRS045826\_contig\_number\_19153SRS049773\_contig\_number\_767SRS049995\_contig\_number\_26428SRS051031\_contig\_number\_12099SRS064276\_contig\_number\_contig-100\_810.221659SRS078176\_contig\_number\_23418SRS078665\_contig\_number\_3239SRS1041139\_contig\_number\_337SRS140645\_contig\_number\_265SRS142542\_contig\_number\_13729SRS147653\_contig\_number\_6532SRS148196\_contig\_number\_contig-100\_686.242952SRS892882\_contig\_number\_12463SRS893170\_contig\_number\_259SRS893373\_contig\_number\_26601
species
658662
20


SRS075878\_contig\_number\_22496
1
species
387661

2293117
species
4

SRS021948\_contig\_number\_31106SRS062701\_contig\_number\_5186SRS1041142\_contig\_number\_6567SRS1055038\_contig\_number\_2462

species
46503
5

SRS049712\_contig\_number\_25111SRS1041037\_contig\_number\_11476SRS104400\_contig\_number\_36775SRS105082\_contig\_number\_11340SRS143876\_contig\_number\_21320

family
171552
4

genus
577309
4


SRS014415\_contig\_number\_contig-100\_112.31009SRS050299\_contig\_number\_1235SRS077086\_contig\_number\_14571SRS147766\_contig\_number\_32
4
454154
species

family
1853231
2

1926651
genus
1

1841857
species
1

SRS046717\_contig\_number\_138

genus
574697
1

1
species
2042309

SRS143181\_contig\_number\_contig-100\_680.89679
